# *RsERF40* contributes to cold stress tolerance and cell expansion of taproot in radish (*Raphanus sativus* L.)

**DOI:** 10.1093/hr/uhad013

**Published:** 2023-02-01

**Authors:** Cui Li, Baozhen Mao, Kai Wang, Liang Xu, Lianxue Fan, Yan Wang, Ying Li, Yinbo Ma, Lun Wang, Liwang Liu

**Affiliations:** National Key Laboratory of Crop Genetics & Germplasm Enhancement and utilization, Key Laboratory of Horticultural Crop Biology and Genetic Improvement (East China) of MOAR, College of Horticulture, Nanjing Agricultural University, Nanjing 210095, China; National Key Laboratory of Crop Genetics & Germplasm Enhancement and utilization, Key Laboratory of Horticultural Crop Biology and Genetic Improvement (East China) of MOAR, College of Horticulture, Nanjing Agricultural University, Nanjing 210095, China; National Key Laboratory of Crop Genetics & Germplasm Enhancement and utilization, Key Laboratory of Horticultural Crop Biology and Genetic Improvement (East China) of MOAR, College of Horticulture, Nanjing Agricultural University, Nanjing 210095, China; National Key Laboratory of Crop Genetics & Germplasm Enhancement and utilization, Key Laboratory of Horticultural Crop Biology and Genetic Improvement (East China) of MOAR, College of Horticulture, Nanjing Agricultural University, Nanjing 210095, China; National Key Laboratory of Crop Genetics & Germplasm Enhancement and utilization, Key Laboratory of Horticultural Crop Biology and Genetic Improvement (East China) of MOAR, College of Horticulture, Nanjing Agricultural University, Nanjing 210095, China; National Key Laboratory of Crop Genetics & Germplasm Enhancement and utilization, Key Laboratory of Horticultural Crop Biology and Genetic Improvement (East China) of MOAR, College of Horticulture, Nanjing Agricultural University, Nanjing 210095, China; National Key Laboratory of Crop Genetics & Germplasm Enhancement and utilization, Key Laboratory of Horticultural Crop Biology and Genetic Improvement (East China) of MOAR, College of Horticulture, Nanjing Agricultural University, Nanjing 210095, China; College of Horticulture and Landscape Architecture, Yangzhou University, Yangzhou 225009, China; College of Horticulture and Landscape Architecture, Yangzhou University, Yangzhou 225009, China; National Key Laboratory of Crop Genetics & Germplasm Enhancement and utilization, Key Laboratory of Horticultural Crop Biology and Genetic Improvement (East China) of MOAR, College of Horticulture, Nanjing Agricultural University, Nanjing 210095, China; College of Horticulture and Landscape Architecture, Yangzhou University, Yangzhou 225009, China

## Abstract

The growth and development of taproots are inhibited by cold stress in radish (*Raphanus sativus* L.). Ethylene-responsive element binding factors (ERF) are key participators in the cold stress response and growth regulation of plants. However, the function of *ERF* genes in cold tolerance and root development in radish remains elusive. Here, we showed that the secondary growth of radish taproots was inhibited by cold stress. Comparative transcriptome analysis demonstrated that the *RsERF40* gene is an important regulator of the cold stress response and root growth regulation. The cold tolerance of transgenic *Arabidopsis* plants overexpressing the *RsERF40* gene was significantly improved. Overexpressing *RsERF40* in the cold-sensitive radish genotype and silencing *RsERF40* in the cold-tolerant radish genotype indicated that *RsERF40* was beneficial for alleviating oxidative damage under cold stress in radish. Transgenic *Arabidopsis* seedlings showed an increase in the elongation and radial growth of dark-grown roots. RT-qPCR analysis showed that the expression of the cold-related genes (CORs) *RsCOR78* and *RsCOR413PM1* and the cell wall strengthening-related genes *RsCESA6* and *RsEXPB3* was upregulated in transgenic *Arabidopsis* seedlings. Yeast one-hybrid (Y1H) and dual-luciferase reporter assays (DLA) revealed that RsERF40 directly regulates *RsCOR78*, *RsCOR413PM1*, *RsCESA6* and *RsEXPB3* expression, illustrating that RsERF40 enhances cold tolerance and taproot growth by modulating osmotic adjustment and cell wall mechanical strength in radish. In this study, the RsERF40-regulon was firstly found to be a new cold response pathway independent of the CBF-COR pathway conferring cold stress tolerance with increasing radish taproot growth. These results provided novel insight into the molecular mechanism underlying cold stress response and would facilitate the genetic improvement of cold tolerance in radish and other root vegetable crops.

## Introduction

Cold stress can be classified as chilling (0–15°C) and freezing (<0°C) stress, impairing the growth of plants, which not only restricts the cultivation area and season but also reduces the crop yield [1]. The damage brought by low temperature stresses on crop production is gradually increased as a consequence of the climate change and temperature fluctuations. Freezing induces ice crystals in the cell wall causing cellular dehydration stress, while chilling stress causes injuries associated with metabolic imbalance [2]. Plants stimulate a series of physiological and biochemical reactions to endure the cold stress. For instance, plants can generate more proline accumulation to maintain osmotic equilibration and trigger significant increases in oxidoreductase activity to prevent oxidative damage [3]. Nevertheless, cold stress response is a complex, tightly orchestrated process and regulated by an intricate transcriptional network, the molecular mechanism underlying cold stress response in plants needs to be fully interpreted.

Transcriptome analysis has provided useful support for elucidating the molecular constituents of cold stress response in several plant species, including *Arabidopsis* [4], rice [5], and *Brassica rapa* L. [6]. With the development of sequencing, bioinformatics approaches and biotechnology, increasing evidences indicate that the ERFs play essential roles in the cold stress response of plants[7]. DREB1s/CBFs (dehydration-responsive-element-binding 1s/C-repeat binding factors) belong to the ERF family and comprise the core of cold signal transduction in plants [8]. DREB1s/CBFs regulate the expression of *CORs* (cold-regulated genes) via direct binding to the CRT (C-repeat element: RCCGAC, R = A/G) in the promoters of *CORs* and enhance the cold tolerance [1]. The cold tolerance of transgenic *Arabidopsis* plants is significantly improved via overexpressing a *DREB1/CBF* from *Arabidopsis* and other plant species, including rice, tomato, and barley, which suggests that the function of *DREB1/CBF* genes is evolutionarily conserved in higher plants [9]. Other *ERF* genes, apart from *DREB1s/CBFs* have been identified as playing crucial roles in the response of plantsto cold stress. For example, *ERF102* and *ERF103* are required for a cold acclimation response in *Arabidopsi*s [10]. For woody plants, overexpressing *BpERF13* enhances the cold tolerance of birch [11]. In fruit trees, the *MdERF1B* gene positively regulates the coldtolerance of apple [12]. However, research on elucidating the cold response molecular mechanism mainly focuses on leaves and fruit rather than roots.

In addition, increasing evidences have assigned a major anddiversified role in plant growth regulation to the ERF family. Overexpression of *CBF* genes inhibits the growth of *Arabidopsis* plants [2, 9]. In contrast, field trials of *OsDREB1C*-overexpressing rice revealed a substantial yield increase by enhancing photosynthetic capacity and improving nitrogen utilisation, and overexpression of *StDREB1* significantly increased tuber weight in potato [13, 14], indicating that the *ERF* genes from the same subfamily showed diverse regulation mechanisms in different species or tissues. Moreover, several ERF transcription factors involved in the transcriptional network underlying root development and growth have been elucidated. In *Arabidopsis*, ERF71 is involved in root development by binding the *cis*-acting CRT element of root cell expansion genes [15]. Transgenic cassava overexpressing *AtCBF3* shows retarded plant growth and a decline in storage root yield [16].

Radish (*Raphanus sativus* L.) is a Brassicaceae species which is commonly cultivated for its edible taproot. Cell division cycle proteins (CDCs), expansins (EXPs) and xyloglucan endotransglucosylase/hydrolase proteins (XTHs) are responsible for cell division and expansion during the radish taproot thickening process [17]. The root thickening process depends on appropriate temperature conditions, cold stress inhibits the growth and development of taproots. Recently, tissue-specific transcriptome analysis conjectured that *ERF*-*1* is an integrator between environmental sensing and growth in radish [18]. However, the intricate regulatory mechanisms of *ERF* genes in integrating cold stress signals with root development remain largely unknown for radish. In this study, a cold-induced ERF gene, *RsERF40*, was identified with comparative transcriptome analysis. The transient expression assay indicated that *RsERF40* contributes to the alleviation of oxidative damage under cold stress in radish. *RsERF40* overexpression enhanced cold tolerance and promoted root growth of *Arabidopsi*s plants. Further analysis confirmed that RsERF40 directly activated the expression of *RsCOR78*, *RsCOR413PM1*, *RsCESA6*, and *RsEXPB3*, demonstrating that RsERF40 participates in regulating the cold stress response and cell expansion of taproots in radish. These results provided peculiar insight into the function of *ERF* genes in regulating the cold stress response and root growth of radish, and will greatly contribute to generating cold stress-tolerant germplasm with higher yields in radish and other root vegetables crops.

## Results

### Cold stress inhibits the secondary growth of radish taproots

**Figure 1:**
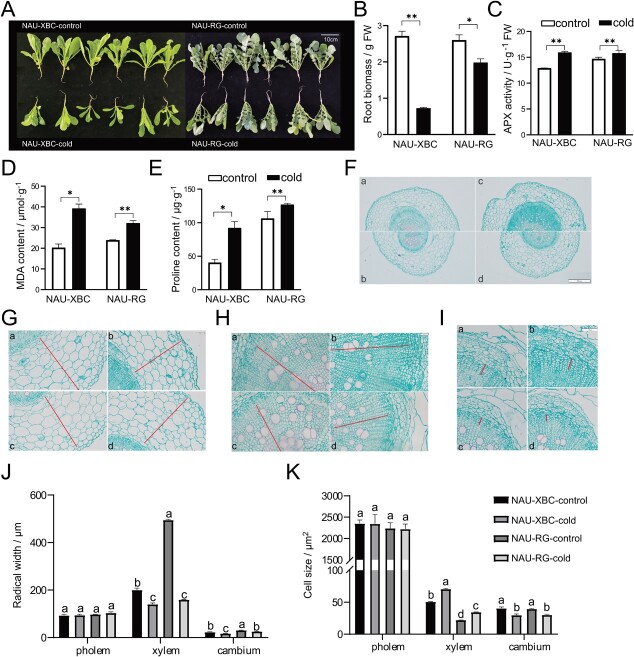
The effect of cold stress on the growth of radish taproots with different cold tolerance. **A** Phenotypic characteristics of radish genotype ‘NAU-XBC’ and ‘NAU-RG’ after cold stress. **B** Root biomass. **C** APX activity. **D** MDA content. **E** Proline content. **F** Representative cross-section images of taproot. Scale bar = 200 μm. **G**–**I** Observation of phloem (**G**, scale bar = 50 μm), xylem (**H**, scale bar = 20 μm) and cambium (**I**, scale bar = 10 μm), a: NAU-XBC-control, b: NAU-XBC-cold, c: NAU-RG-control, d: NAU-RG-cold. **J** Radial width of phloem, xylem, and cambium zone. **K** Size of the cell in phloem, xylem, and cambium. Lowercase letters indicate statistically significant differences based on one-way ANOVA with Duncan’s multiple range test; the asterisk(s) indicate significant differences based on Student’s *t* test (^*^P < 0.5, ^**^P < 0.01).

Two radish advanced inbred lines, ‘NAU-XBC’ and ‘NAU-RG’, were treated with cold stress to investigate their phenotypic changes. Cold stress significantly inhibited the growth and development of radish taproots (Fig. 1A). In the morphological and physiological characteristics assay, taproot biomass decreased by 62.7% and 22.1% in ‘NAU-XBC’ and ‘NAU-RG’ after cold treatment, respectively (Fig. 1A and B). The activity of APX (ascorbate peroxidase) significantly increased under cold stress in both radish genotypes (Fig. 1C). Compared with the control, MDA accumulation increased by 95.1% in ‘NAU-XBC’ and 36.8% in ‘NAU-RG’ under cold stress (Fig. 1D), while proline content in ‘NAU-XBC’ was less than that in ‘NAU-RG’ (Fig. 1E). These results indicated that ‘NAU-XBC’ was a cold-sensitive genotype, while the ‘NAU-RG’ was cold-tolerant. Further observation revealed that the radial width of the phloem zone and the cell size in the phloem zone exhibited insignificant differences, while the radial width of the cambium and xylem zone was decreased under cold stress (Fig. 1F–K). The cell size in the cambium zone was reduced, suggesting that the developmental process of cambium cells was restrained under cold stress. The size of parenchyma cells in the xylem zone increased, indicating that the number of the parenchyma cells decreased after cold stress treatment (Fig. 1K). These results demonstrate that cold stress severely inhibited secondary growth and reduced radish taproot production.

### Cold-induced transcriptomic changes between the two radish genotypes

**Figure 2:**
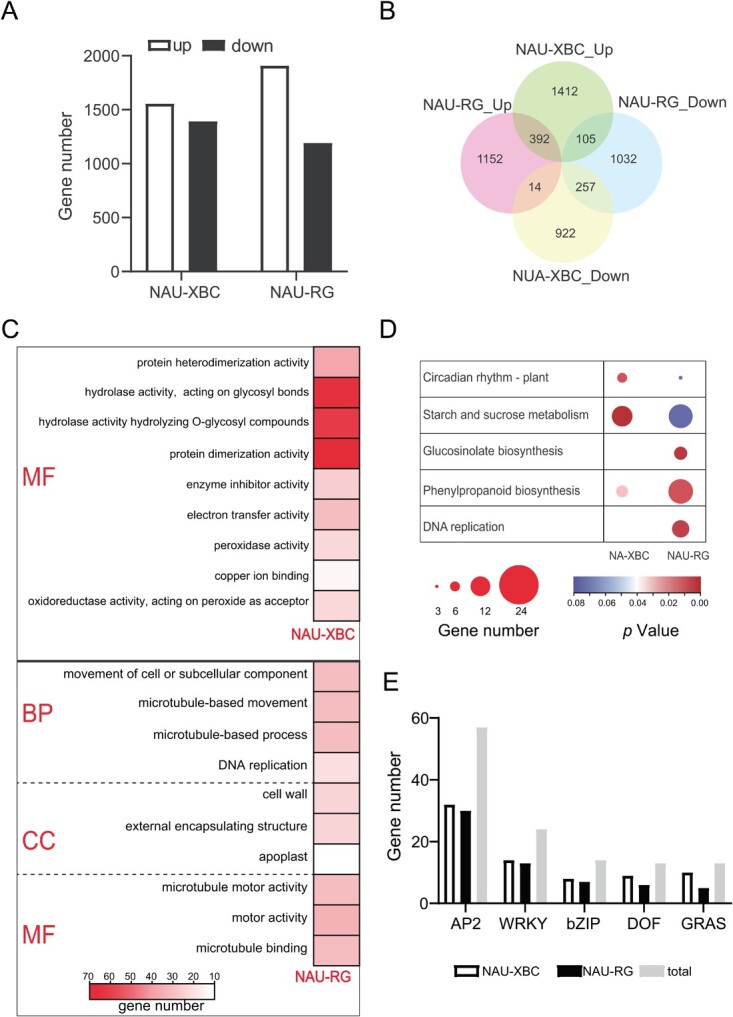
Transcriptome analysis of two radish genotypes under cold stress. **A** The number of DEGs. **B** Venn diagram of DEGs in different genotype radish (‘NAU-XBC’: cold sensitive genotype and ‘NAU-RG’: cold tolerant genotype). **C** GO enrichment; **D** KEGG analysis. **E** The number of transcription factors that were differentially expressed under cold stress.

To explore the transcriptional regulation mechanism of radish taproots under cold stress, 12 cDNA libraries were constructed from radish taproots treated with cold and normal temperature conditions (Table S1, see online supplementary material). The Pearson’s correlation and principal component analysis showed that all biological replicates had a strong correlation, and samples from each treatment belonged to the same cluster with a similar pattern (Fig. S1A and B). Moreover, the expression patterns of nine randomly selected genes from RT-qPCR fit well with those from RNA-seq data. These results indicated that the RNA-seq data were reliable (Fig. S1C). As a result, 2952 and 3102 differentially expressed genes (DEGs) were identified from ‘NAU-RG’ and ‘NAU-XBC’, respectively (Fig. 2A). Among them, less than 18% were commonly regulated, while more than 74% of the DEGs were specifically regulated in the two genotypes (Fig. 2B). GO enrichment analysis showed that nine molecular function (MF) categories were enriched in the DEGs of ‘NAU-XBC’, and the DEGs in ‘NAU-RG’ were significantly enriched in 10 GO terms, comprising four biological processes (BP), three cellular components (CC), and three MF categories (Fig. 2C). KEGG analysis demonstrated that DEGs in ‘NAU-XBC’ were significantly enriched in ‘Starch and sucrose metabolism’ and ‘Circadian rhythm – plant’, while the significantly enriched pathways of DEGs in ‘NAU-RG’ were ‘glucosinolate biosynthesis’, ‘phenylpropane biosynthesis’ and ‘DNA replication’ (Fig. 2D). Further analysis indicated that cold stress had the opposite effect on these DEGs related to the ‘glucosinolate biosynthesis’ and ‘DNA replication’ pathways in ‘NAU-XBC’ and ‘NAU-RG’ radish genotypes (Fig. S2), suggesting that the upregulation of both pathways specifically plays critical roles in the cold tolerance of radish taproots.

The expression profiles of key TFs potentially involved in the cold stress response (ERF, WRKY, bZIP, DOF, and GRAS) were explored (Table S2, see online supplementary material). In ‘NAU-XBC’, the expression of 32 *ERFs*, 14 *WRKYs*, 8 *bZIPs*, 9 *DOFs*, and 10 *GRASs* showed significant differences under cold stress, the number was more than in ‘NAU-RG’. A total of 57 *ERF*, 24 *WRKY*, 14 *bZIP*, 13 *DOF*, and 15 *GRAS* genes were induced by cold stress (Fig. 2E), indicating that cold stress has a great influence on the expression of *ERF* genes in radish taproots. Further analysis revealed that ‘NAU-XBC’ and ‘NAU-RG’ shared five differentially expressed *ERF* genes under cold stress, suggesting that ERF TFs might play important roles in the response of radish taproots to cold stress.

### *RsERF40* functions as a positive regulator in the cold stress response

**Figure 3:**
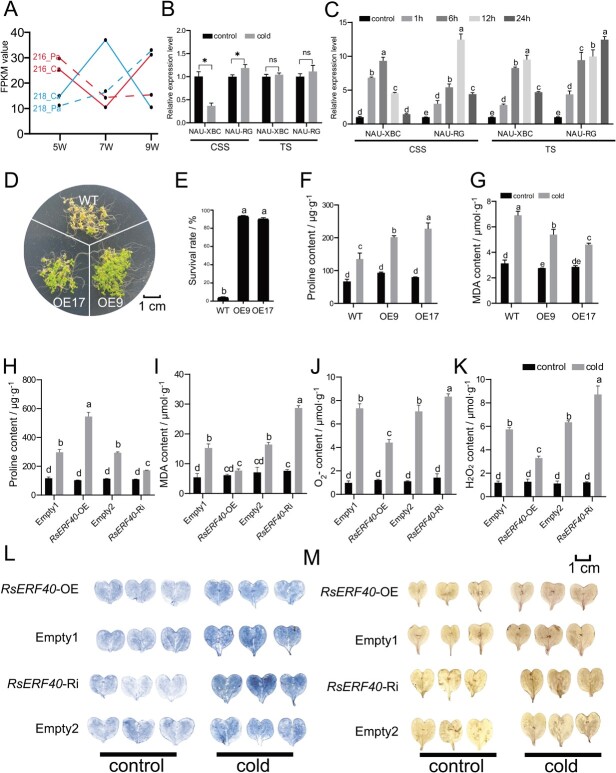
*RsERF40* positively regulates the cold tolerance of plants. **A** The FPKM value of *RsERF40* during the taproot development in radish genotype 216 with large taproot and genotype 218 with small taproot, the transcriptome data were obtained from Hoang *et al.* [18]. **B** and **C** RT-qPCR assay of *RsERF40* in two radish genotypes after long-term (**B**) and short-term (**B**) cold treatment. CS: cortex splitting stage, TS: thickening stage. **D** Freezing treatment. **E** The survival rate of *Arabidopsis* plants after freezing treatment. **F** and **G** Proline (**F**) and MDA (**G**) content in *Arabidopsis* plants. **H**−**K** Proline (**H**), MDA (**I**), O_2_^−^ (**J**), and H_2_O_2_ (**K**) content in radish cotyledons. The *RsERF40-*OE represents the cotyledons overexpressing *RsERF40* gene and the *RsERF40*-Ri represents the cotyledons silencing of *RsERF40* gene. Empty1 and Empty2 represents the radish cotyledon injected with the pCAMBIA1301 vector and pTCK303 vector, respectively. **L** and **M** NBT (**L**) and DAB (**M**) staining of radish cotyledons. The asterisk(s) indicate significant differences based on Student’s t test (^*^P <0.5, ^**^P< 0.01). Lowercase letters indicate statistically significant differences based on one-way ANOVA with Duncan’s multiple range test.

The cold-induced expression levels of *ERF* genes during taproot growth were investigated using the available tissue- and stage-specific transcriptome data (Fig. S3A, see online supplementary material). In the cambium and xylem, *Rsa4g016700* expression showed opposite trends during taproot development of radish genotype 216 with a large taproot and genotype 218 with a small taproot [18], indicating that *Rsa4g016700* plays a critical role in the developmental regulation of radish taproots (Fig. 3A). RT-qPCR was used to address the possibility that differences in plant genotype and stage accounted for the differences in *Rsa4g016700* expression levels under cold stress. The expression level of *Rsa4g016700* was decreased in ‘NAU-XBC’, whereas it was increased in ‘NAU-RG’ after long-term cold stress at the cortex splitting stage (CSS) (Fig. 3B). After cold stress for 7 d at the thickening stage (TS), the *Rsa4g016700* gene remained relatively steady in the taproots of ‘NAU-XBC’ and ‘NAU-RG’ (Fig. 3B). In addition, the expression level of the *Rsa4g016700* gene at 0, 1, 3, 6, 12, and 24 h after the switch in cold stress was determined to investigate the dynamic changes of the *Rsa4g016700* gene during cold treatment. The expression level of *Rsa4g016700* initially increased at 1 h and reached the maximum level at 6 h (CSS) or 12 h (TS) in ‘NAU-XBC’, while the *Rsa4g016700* expression increased from 1 h to 12 h (CSS) or 24 h (TS) after cold treatment in the taproot of ‘NAU-RG’ (Fig. 3C). These results indicated that *Rsa4g016700* was regulated by cold stress and the greater *Rsa4g016700* expression was performed during short-term cold treatment. Therefore, *Rsa4g016700* was recognised as a key candidate gene involved in regulating the cold stress response during radish taproot development. Subsequently, a 675-bp open reading frame (ORF) of *Rsa4g016700* was isolated from ‘NAU-RG’ and encoded a protein of 224 amino acids (aa) with an AP2 domain. Sequence alignment analysis by Blastp showed that *Rsa4g016700* had the highest similarity with *AtERF40*, belonging to the TINY-like class in the ERF family and was named RsERF40 (Fig. S3B). Alignment of the AP2 domain of ERF40 showed that the 14th and 19th amino acids responsible for binding to CRT were completely consistent between radish and *Arabidopsis* (Fig. S3C), suggesting that RsERF40 may have the ability to bind to CRT. The 51st aa was diverse in different plant species and was threonine (T) only in radish, which might be related to the exclusive function of RsERF40 in radish.

To explore the function of *RsERF40* in the cold stress response, two transgenic *Arabidopsis* lines overexpressing *RsERF40* (OE9 and OE17) with different expression levels were generated via *Agrobacterium*-mediated transformation (Fig. S4A and B). After exposure to 1.0 h freezing shock (−5°C) followed by 5 d recovery at room temperature, the survival rate of *RsERF40* transgenic *Arabidopsis* significantly increased compared to WT plants (Fig. 3D and E). Under cold stress, *RsERF40-*OE plants accumulated less MDA and more proline than the WT plants (Fig. 3F and G). To further investigate the function of *RsERF40* in radish, *RsERF40-*OE and *RsERF40*-Ri radish cotyledons were obtained by transient transfection (Fig. S4C). Compared with control plants, *RsERF40-*OE plants produced less MDA and more proline after cold treatment, while the *RsERF40-*Ri plants showed contrasting phenotypes (Fig. 3H and I). Moreover, the accumulation of O_2_^−^ (NBT staining) and H_2_O_2_ (DAB staining) in cotyledons was markedly decreased in *RsERF40-*OE plants, while both were increased in the *RsERF40-*Ri plants after cold stress treatment (Fig. 3J−M), suggesting that *RsERF40* alleviates the oxidative damage and osmotic injury caused by cold stress.

### RsERF40 regulates cold tolerance via a CBF-independent pathway

**Figure 4:**
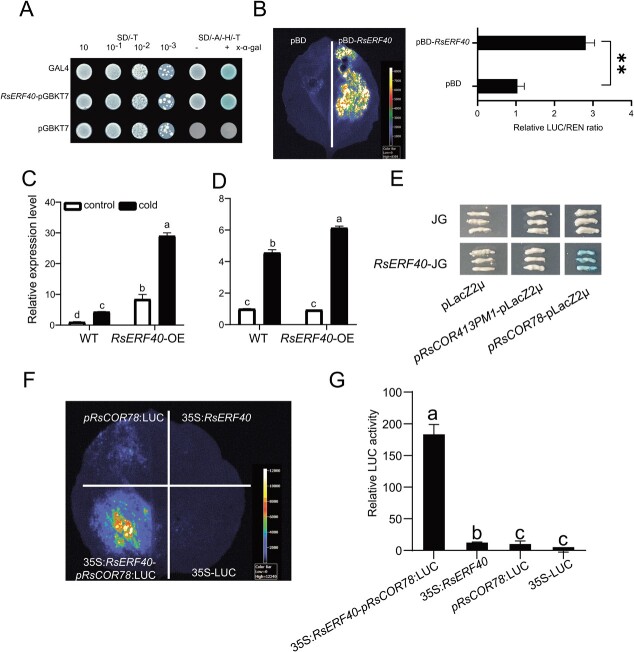
RsERF40 regulates the cold tolerance through directly activating the expression of *RsCOR78*. **A** and **B** Transcriptional activity analysis of RsERF40 protein in yeast (**A**) and tobacco leaves (**B**). **C** and **D** RT-qPCR analysis of *COR78* (**C**) and *COR413PM1* (**D**) in *Arabidopsis.* (**E**) Y1H (Yeast one hybrid). **F** and **G** LUC fluorescence imagining (**F**) and DLA (Dual luciferase reporter assay) (**G**) in tobacco leaves co-transformed by the reporter (*pRsCOR78*:LUC) and the effector (35S:*RsERF40*). The asterisk(s) indicate significant differences based on Student’s t test (^*^P < 0.5, ^**^P < 0.01). Lowercase letters indicate statistically significant differences based on one-way ANOVA with Duncan’s multiple range test.

The transcriptional ability of RsERF40 was investigated to explore the regulation mechanism of RsERF40 in the cold stress response. The yeast cells harbouring *RsERF40*-pGBKT7 showed α-gal activity in SD/−Trp/-His/−Ade, and pBD-*RsERF40* had significantly stronger fluorescence activity than pBD-Empty in tobacco leaves (Fig. 4A and B), indicating that RsERF40 possessed transcriptional activity. Y1H was performed to specify the target sequence of RsERF40 using CRTs (RCCGAC) and single-base-substituted mCRTs (RCCGAC) fragments. The ‘RCCGAC’ sequence was specifically recognised and bound by RsERF40 (Fig. S5A). *Cis*-element analysis found that *RsCOR78* and *RsCOR413PM1* had CRTs in their promoters. The expression levels of *COR78* and *COR413PM1* significantly increased in *RsERF40-*OE plants under normal conditions. Under cold stress, the *COR78* expression level increased by 5.7 times more in *RsERF40-*OE plants than in WT plants (Fig. 4C and D), suggesting that RsERF40 directly regulated *COR78* expression. The Y1H assay showed that only the yeast that transformed both *pRsCOR78*-lac and *RsERF40*-JG could turn blue in the SD/−Trp/-Ura medium with X-gal (Fig. 4E). The DLA revealed that RsERF40 increased the promoter activity of *pRsCOR78* (Fig. 4E and G) Fig. 4E and F, indicating that RsERF40 activates *RsCOR78* expression.

### RsERF40 directly binds to cell wall strengthening-related genes *RsCESA6* and *RsEXPB3*

**Figure 5:**
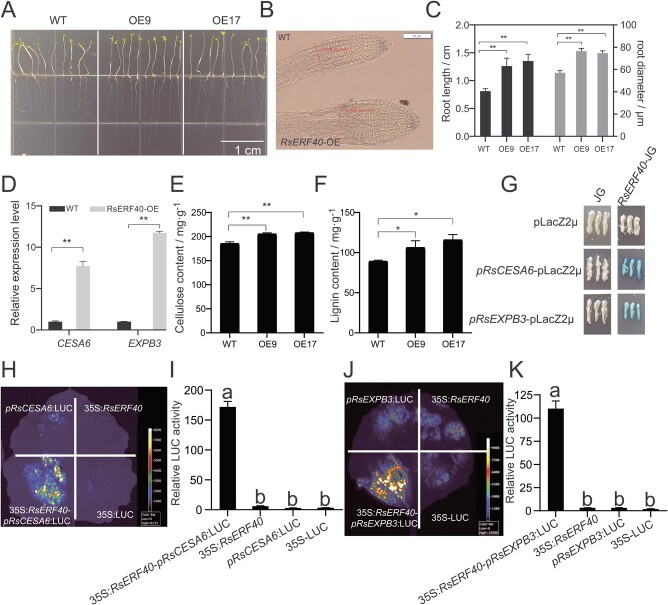
RsERF40 regulates taproots growth through regulating the expression of *RsCESA6* and *RsEXPB3*. **A** Observation of the *Arabidopsis* plants cultured in the MS medium. **B** Observation of the root of *Arabidopsis* plants using light microscope. **C** Statistical analysis of the root length and diameter of *Arabidopsis* plants. **D** Expression analysis of *RsCESA6* and *RsEXPB3* in root of transgenic *Arabidopsis* plants grown in the dark. **E** and **F** Cellulose (**E**) and lignin (**F**) content in the root of *Arabidopsis* plants cultured in the dark. (**G**) Y1H. **H** and **K** LUC fluorescence imagining (**H** and **J**) and DLA (**I** and **K**) in tobacco leaves. The asterisk(s) indicate significant differences based on Student’s t test (^*^P < 0.5, ^**^P < 0.01). Lowercase letters indicate statistically significant differences based on one-way ANOVA with Duncan’s multiple range test.

*Arabidopsis* plants were cultured in dark conditions for 7 d to ascertain whether *RsERF40* was involved in the regulation of root growth in the dark. Compared with wild-type *Arabidopsis*, transgenic *Arabidopsis* lines displayed a significantly larger root length and root diameter of 30–33% (Fig. 5A–C), indicating that *RsERF40* promoted root growth in *Arabidopsis*. Among a total of 27 genes identified to be related with taproot development according to transcriptome sequencing data, *cis*-element analysis found that promoters of seven genes, *RsCSLC5*, *RsCDC5*, *RsXTH7*, *RsXTH9*, *RsEXPB3*, *RsEXPA12*, and *RsCESA6*, had at least one CRT element (RCCGACA). In particular, there were four and three CRT elements in the promoter of the cellulose biosynthesis gene *RsCESA6* and β-expansin gene *RsEXPB3*, respectively (Table S3, see online supplementary material). After culturing in the dark for 7 d, *CESA6* and *EXPB3* expression showed higher levels in *RSERF40*-OE than in WT plants (Fig. 5D). Significantly larger amounts of cellulose and lignin were accumulated in the *RsERF40*-OE *Arabidopsis* plants than in the WT (Fig. 5E and F). Therefore, it could be inferred that *RsERF40* controls the root development by regulating the expression of *RsCESA6* and *RsEXPB3* genes. Subsequently, Y1H analysis revealed that the promoters of *RsCESA6* and *RsEXPB3* were bound by RsERF40 (Fig. 5G). DLA confirmed that the LUC signal and activity were significantly increased when *RsERF40–*1300 was co-expressed with *pRsCESA6*–0800 or *pRsEXPB3*–0800 (Fig. 5H–K), implying that RsERF40 could directly promote *RsCESA6* and *RsEXPB3* expression, and these findings demonstrated that RsERF40 was involved in root development regulation by promoting the expression of *RsCESA6* and *RsEXPB3*.

**Figure 6:**
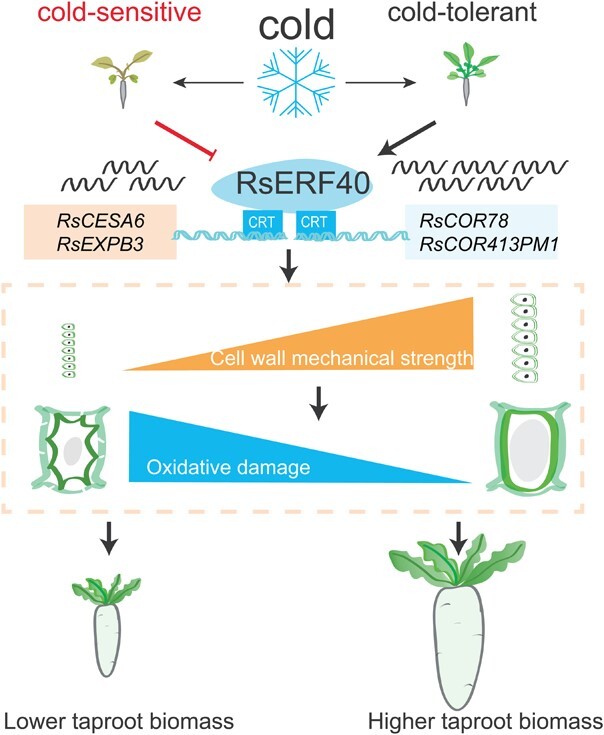
RsERF40 plays a positive role in cold stress response and cell expansion of taproots in radish. RsERF40 activates the expression of *RsCOR78*, *RsCOR413PM1*, *RsCESA6*, and *RsEXPB3* that enhances the cold tolerance and the cell expansion of taproot in radish. The difference of transcription level of *RsERF40* between two radish genotypes under long-term cold stress differentiates the cold tolerances. The black curved lines represent the transcript of *RsERF40* gene, and the blue curved lines indicate the promoters of genes.

## Discussion

Cold stress inhibits plant growth and limits the cultivation time and geographical distribution of plants [1]. The ERF family has been reported as a regulator of plant growth and cold stress response in many plant species [7]. However, information on ERF TFs in radish is limited. In this study, the regulatory mechanism of RsERF40 in the cold stress response and root development was comprehensively investigated.

### RsERF40 advocates the radish taproot growth by regulating cell expansion

Because the taproot is the important edible organ of radish, it is worthwhile to explore the mechanism of cold stress affecting root growth and development in radish. Scattered studies have revealed that *ERF* genes are involved in regulating underground tissue growth, such as potato [14] and cassava [16]. In the present study, a contrasting trend for *RsERF40* expression during taproot growth was shown between two radish genotypes with different root sizes, and the roots of *RsERF40*-OE *Arabidopsis* were stronger than those of the WT, indicating that *RsERF40* was positively associated with taproot development. The *tiny* mutant plants overexpressing the *TINY* gene had a de-etiolated response and radial thickening of the hypocotyl in dark conditions [19]. Overexpressing the potato *TINY-like* gene *StDREB1* significantly increased the tuber weight in potato [14]. The root growth of *SlDREB3-*overexpressing tomato plants increased by 50% more than that of wild-type [20]. These results demonstrate that *RsERF40* has a beneficial effect on the growth of underground tissues.

The cell size and shape were determined by the plant cell wall via the mechanical control of cell expansion. Cell wall biosynthesis is closely associated with cell expansion, which plays a crucial role in root development [21]. The cellulose and lignin content significantly increased in *RsERF40*-OE roots in the dark, and the expression level of *AtCESA6* was upregulated. CESA6 encoded a cellulose synthase isomer, and loss of function *procuste1* alleles of *CESA6* showed inhibition of hypocotyl and root growth in dark-grown seedlings. However, *AtCESA6* overexpression greatly enhances plant growth by increasing cell growth and cell wall thickness in *Arabidopsis* plants [22, 23]. This study confirmed that there was a protein-DNA interaction between RsERF40 and *RsCESA6*, indicating that RsERF40 regulated cell growth and the cell wall thickness of radish taproots. *TINY-like* genes positively regulate primary cell wall-type *CESA* genes to adjust the primary and secondary cell walls biosynthesis [24]. These results suggest that RsERF40 promotes the cellulose synthase to accelerate cell expansion in radish taproots.

Expansins are cell wall-related proteins that participate in cell enlargement and expansion through regulating the cell wall extensibility [25]. Co-expression network analysis of different ploidy Ma bamboo revealed that *EXPB3* had a potential regulatory role in regulating cell wall expansion [26], and transcriptomic profiling insights into the dynamic regulation of taproot growth found that *RsEXPB3* was involved in regulating taproot formation of radish [17]. *Cis*-element analysis showed that the *RsEXPB3* promoter had three CRT elements, and Y1H verified that RsERF40 activates *RsEXPB3* expression. The expression level of *EXPB3* was increased in cortical cells of roots by ethylene treatment in maize [27], indicating that *RsEXPB3* expression was closely associated with ethylene response TFs. These results demonstrated that RsERF40 promotes radish taproot growth by activating *RsEXPB3* expression.

### RsERF40 positively regulates cold tolerance by stabilising cellular osmotic potential and regulating cell wall mechanical strength

Several ERF TF subfamilies including DREB1 (A1 type) and DREB2 (A2 type) [7] were extensively reported to be involved in the cold stress response of plants. In this study, overexpression of the A4 type DREB gene *RsERF40* in *Arabidopsis* and radish revealed that *RsERF40* enhances the cold tolerance of plants. Under cold stress, CBFs specifically activated the expression of *CORs*, which repaired cold-rigidified membranes and stabilised cellular osmotic potential [8]. This study found that there was more free proline accumulation and a decrease in MDA content in the *RsERF40-*OE plants, and the expression of the *COR413PM1* and *COR78* genes showed a significant increase in *RsERF40-OE* plants. *Cis*-element analysis revealed one and two CRT elements in the promoters of *RsCOR413PM1* and *RsCOR78,* respectively. However,RsERF40 specifically recognised and bound to the CRT element in the promoter of *RsCOR78* but not *RsCOR413PM1* in the Y1H system using the *LacZ* gene as a reporter. It was speculated that the binding between RsERF40 and the *RsCOR413PM1* promoter was too weak to be detected by the EGY48-*pLacZ* system.Therefore, the Y187-*pHis2* yeast one-hybrid system and DLA were used to explore the interaction between RsERF40 and the *RsCOR413PM1* promoter. The results showed that RsERF40 activated *RsCOR413PM1* expression (Fig. S5B and C). *COR413PM1* encoded a multi-spanning transmembrane protein and affected the metabolism of fatty acids, sugars, and purine to regulate the ability of osmotic adjustment under cold stress in *Arabidopsis* [28]. *COR78* is an osmotically responsive gene induced by cold stress and is important for root growth at low temperatures in *Arabidopsis* [29]. In general, *COR78* and *COR413PM* were regulated by CBFs [3], while RsERF40 enhanced the cold tolerance by directly activating the expression of *RsCORs* in this study. There are two conserved signature motifs in the CBF proteins PKKPAGR (RAGRxxKFxETRHP) and DSAWR, and the PKKPAGR motif is important for the biological function of CBF proteins [30, 31]. The comparison of amino acid sequences showed that neither of the two signature motifs of CBF proteins was found in RsERF40 (Fig. S6, see online supplementary material), indicating that RsERF40 positively regulated cold tolerance through a CBF-independent pathway in plants.

Drastic cold exposure induces extracellular ice formation, and cell wall rigidity is a necessary factor in the cell resistance to cold stress [27, 32]. The cell wall polysaccharide composition and activities of cell wall-modifying enzymes were changed to cope with cold stress in plants [27]. In this study, cold treatment significantly enhanced *RsCESA6* expression in radish taproots (Fig. S7A, see online supplementary material), and the cellulose content of radish taproots was increased in both the ‘NAU-XBC’ and ‘NUA-RG’ genotypes under cold stress (Fig. S7B, see online supplementary material). The expression level of *CESA6* increased due to cold stress in *Arabidopsis* and rice, and the *CESA6* mutation affected membrane integrity under salt stress conditions [33, 34]. These results indicate that *CESA6* plays a major role in cellulose deposition in roots under cold stress conditions [34]. The expression level of *RsEXPB3* increased and the lignin content was upregulated after cold stress in radish taproot (Fig. S7C and D, see online supplementary material). *TaEXPB7-B* overexpression in *Arabidopsis* increased lignin and cellulose content and conferred enhanced antioxidant and osmotic regulation in transgenic *Arabidopsis* [35], *EXP* genes were upregulated in *Arabidopsis* during cold stress [36]. Moreover, the transcript level of *EXPB3* was increased by ethylene treatment [27]. These reports suggest that the *EXPB3* gene might be involved in the cold stress response through the ethylene signal pathway. In this study, RsERF40 was firstly verified to activate *RsEXPB3*, implying that *RsEXPB3* might be regulated by ERFs under cold stress. These results indicated that RsERF40 contributed to cold tolerance by maintaining osmotic balance and regulating cell wall mechanical strength.

In conclusion, this study functionally characterised an ERF transcription factor RsERF40, and revealed that it acts as a positive regulator in the cold stress response and cell expansion in radish taproots (Fig. 6). The expression of the *RsERF40* gene in radish taproots increased with the short-term exposure (hours) to cold stress. RsERF40 directly activates the cold-responsive genes *RsCOR78* and *RsCOR413PM1* to promote the accumulation of cryoprotectants and relieve the oxidative damage induced by cold stress, thereby enhancing the cold tolerance of plants. Moreover, the expression of cell wall strengthening-related genes *RsCESA6* and *RsEXPB3* was directly regulated by RsERF40 and led to mechanical property changes in the cell wall, which not only promotes cell expansion of radish taproots, but also maintains cellular structure integration to stabilise cellular osmotic potential under cold stress, which positively regulates the cold tolerance of radish. At the CSS stage, the expression of the *RsERF40* gene in the cold-sensitive radish genotype decreased after long-term cold treatment, leading to a decrease in cold resistance and the inhibition of taproot biomass in radish. These findings provide novel insights into the molecular control of plant responses to cold stress and facilitate the genetic improvement of cold tolerance in radish and other root vegetable crops.

## Materials and methods

### Plant materials and cold treatment

Radish seeds of ‘NAU-XBC’ and ‘NAU-RG’ were germinated in the dark at 25°C for 2 days. Then the germinated seeds were sowed in the plastic pots containing composite soil of peat and vermiculite (3:1, v/v) with a 16 h photoperiod (illumination was 20 000 lx) at approximately 25/16°C (day/night). At the cortex splitting stage (CSS: 30 days after sowing) and thickening stage (TS: 60 days after sowing), the seedlings were cultured in a growth incubator with a photoperiod of 10°C/16 h light (illumination intensity was 20 000 lx) and 5°C/8 h dark for 1 h, 6 h, 12 h, 24 h, and 7 days. Three biological replicates were used with 10 plants for each replicate. Roots were harvested and stored at −80°C.

### Physiological and biochemical measurements

The activity of the ascorbate peroxidase (APX) enzyme was detected according to a previous study [37]. The malondialdehyde (MDA) content was determined by thiobarbituric acid (TBA) [38], and the proline content was detected by the ninhydrin colorimetry method [39]. Radish taproots were dried at 80°C for 3 d to a constant weight, and then the anthrone–sulfuric and ultraviolet spectrophotometry methods were employed to determine the cellulose and lignin content in radish taproots, respectively [40, 41].

### Embedding, sectioning, and staining

The taproots of ‘NAU-XBC’ and ‘NAU-RG’ were collected and soaked in PBS (pH 7.2) containing 2.5% glutaraldehyde at 4°C for 24 h. The specimens were embedded into paraffin blocks which were sectioned at a thickness of 8–10 μm using the Leica thin-sliced cutting machine (Weztlar, Germany) [42, 43]. After histochemical staining, the sections were imaged with an optical microscope (Olympus BX53F).

### RNA-seq and DEG analysis

RNA from the taproots of ‘NAU-XBC’ and ‘NAU-RG’ after 7 d of cold stress at CSS were used for RNA-seq. A total of 12 libraries were generated using Illumina Paired End Sample Prep Kit. The clean reads were aligned to the radish reference genome by HISAT2 (v2.0.5) [44]. The expression level of transcript was determined by FPKM value (fragments per kilobase of exon per million mapped fragments). The genes with *P* < 0.05 and fold change >2 were identified as DEGs by DESeq2 analysis [45]. A total of nine DEGs were randomly selected for RT-qPCR to guarantee the accuracy of the RNA-seq data. The primers used for RT-qPCR are listed in Table S4 (see online supplementary material). The functions of DEGs were explored using the GSEA analysis tool (http://www.broadinstitute.org/gsea/index.jsp) [46] for GO functional classification and KEGG pathway enrichment. The GO terms and KEGG pathways with corrected *P* < 0.05 were defined as significantly enriched.

### Sequence alignment and phylogenetic analysis of RsERF40

The amino acid sequence of RsERF40 was used as a query to perform a BLAST search in NCBI (https://www.ncbi.nlm.nih.gov). The sequences of TINY and CBF proteins were obtained from TAIR (https://www.arabidopsis.org). MEGA X was used to construct a phylogenetic tree based on the neighbour-joining method and bootstrap analysis with 1000 replications [47]. Multiple sequence alignment using Clustal X and displayed using ENDscript [48].

### *RsERF40* overexpression in *Arabidopsis* plants

The complete ORF without the stop codon of the *RsERF40* gene was amplified and ligated into pCAMBIA1301. The *Agrobacterium tumefaciens* strain GV3101 carrying the *RsERF40-*pCAMBIA1301 vector was delivered into *A.thaliana* (Col-0) using the floral-dip method [49]. Transgenic *Arabidopsis* plants overexpressing the *RsERF40* gene were identified by selection on Murashige and Skoog (MS) plates containing 36 mg/L hygromycin, along with semi-qPCR amplification. *Arabidopsis* seeds were planted on MS plates and then grown vertically at 22°C in the dark for 5 d before imaging. The roots of two-week-old *Arabidopsis* seedlings (grown in the dark) were harvested and dried to a constant weight at 80°C for 2 d, and then the content of cellulose and lignin content were detected [40, 41]. For freezing treatment, *Arabidopsis* seedlings were grown at normal condition (22°C with a 16 h light/8 h dark photoperiod, illumination was 20 000 lx) for 5 d, and then were transferred to −5°C or 22°C for 1 h in the dark. After freezing treatment, the seedings were transferred to normal conditions for 5 d to explore the survival rate and detect the MDA and proline content. The RNA of *Arabidopsis* roots was extracted for RT-qPCR analysis.

### Transient expression analysis

To construct the *RsERF40*-RNAi vector, a 382 bp fragment of *RsERF40* was fused into a pTCK303 vector [50]. *A. tumefaciens* strain GV3101 carrying the *RsERF40*-pCAMBIA1301 vector or *RsERF40*-RNAi was injected into cotyledons of ‘NAU-XBC’ and ‘NAU-RG’ according to a reported method, respectively [51, 52]. These radish plants were cultured at 25°C or 4°C with 20 000 lx illumination for 6 h. Fresh cotyledons were collected for physiological and biochemical measurements, nitroblue tetrazolium (NBT) and diaminobenzidine tetrahydrochloride (DAB) staining [53, 54], and the remaining cotyledons were frozen in liquid nitrogen freezing for the RT-qPCR assay.

### Transcriptional activation analysis of RsERF40

The CDS of RsERF40 was inserted into the pGBKT7 vector, and then the fusion construct RsERF40-pGBKT7 was transformed into yeast strain AH109. Yeast cells were serially diluted and spread onto SD/−Trp or SD/−Ade/-His/−Trp medium with 0 or 20 μg/mL a-galactosidase [55] The CDS of RsERF40 was inserted into the constructed pBD vector driven by the 35S promoter and introduced into the *A. tumefaciens* strain GV3101, and then injected into tobacco leaves [56].

### Yeast one hybrid (Y1H)

The entire CDS of *RsERF40* was fused in the pB42D vector to construct the prey. The promoter of *RsCOR413PM1*, *RsCOR78*, *RsCESA6*, *RsEXPB3*, triple-repeat CRT element ‘GCCGAC’ (3 × CRT1) and ‘ACCGAC’ (3 × CRT2), mutation of CRT ‘ACCTAC’ (3 × mCRT1), and ‘GCCTAC’ (3 × CRT2) were ligated into the pLacZ2μ vector as the bait. The recombinant plasmids were co-transferred into yeast EGY48 on SD/-Ura/−Trp medium for 3 d at 30°C. The three positive clones were transformed to SD/-Ura/−Trp medium supplemented with 20 μg/mL X-α-gal to determine the protein-DNA interaction based on the β-galactosidase activity (X-gal) [57].

RsERF40 was ligated into the pGADT7 vector and the promoter of *RsCOR413PM1* was fused into the pHIS2 vector. The recombinant vectors were co-transformed into the yeast strain Y187 growing on SD-Trp/-His medium for 3d. For interaction screening, the yeast cells were then transferred to SD-Trp/−Leu/-His medium with 40 μM or without 3-amino-1, 2, 4-triazole (3-AT) [58].

### Dual luciferase reporter assay (DLA)

The pCAMBIA1301-*RsERF40* was used as an effector, CRT, mCRT, the promoter of *RsCOR78*, *RsCOR413PM1*, *RsCESA6*, or *RsEXPB3* was inserted into pGreenII 0800-LUC to generate reporter, respectively. The recombinant vectors were transformed into *Agrobacterium* strain GV3101 to inject into tobacco leaves, and then the tobacco plants were transferred into the dark for 48 h [56]. The Vazyme Dual Luciferase Reporter Assay System (Nanjing, China) was used to analyse the fluorescence activity [52].

### Semi-and RT-qPCR assay

The total RNA from radish and *Arabidopsis* roots was extracted using the Tiangen RNAprep Pure Plant Kit (Beijing, China) [37]. The Vazyme PrimeScript™ RT reagent kit with gDNA Eraser was used for synthesising the first-strand cDNA. *RsActin* and *AtActin* were used as reference genes. The Vazyme Green Taq Mix was used for semi-qPCR according to the manufacturer’s instructions. The Vayme SYBR Green PCR Master Mix was employed for RT-qPCR on a Roche LightCycler 480 System [37]. The 2^−ΔΔ*C*^_T_ method was used for calculating the relative expression level of the target gene [59, 60]. Three independent biological replicates and three technical replicates were performed for each sample. All the primers used in this study are provided in Table S4 (see online supplementary material).

### Statistical analysis

All experiments were carried out at least in triplicate. SPSS 13.0 was used for evaluating the statistical significance by ANOVA. GraphPad Prism 8 was used for the statistical analyses and data visualization.

## Acknowledgments

This work was funded by grants from the National Key Technology R&D Program of China (2018YFD1000800), National Natural Science Foundation of China (32172579), Jiangsu Seed Industry Revitalization Project[JBGS(2021)071], the earmarked fund for Jiangsu Agricultural Industry Technology System [JATS(2022)463], Jiangsu Agricultural Science and Technology Innovation Fund (CX (21)2020) and the Project Founded by the Priority Academic Program Development of Jiangsu Higher Education Institutions (PAPD).

## Author contributions

C.L. and L.L. conceived and designed the research. C.L. and B.M. performed the data analysis and wrote the paper. C.L., K.W., and L.F. performed experiments. L.X., Y.W., and Y.M. contributed powerful analytical tools. L.L., L.W., and Y.L. critically reviewed the manuscript. All authors read and approved the final paper.

## Data availability statement

The raw data of RNA-seq was deposited in the Genome Sequence Archive (GSA) database of the National Genomics Data Center (NGDC) with BioProject ID PRJCA011633.

## Conflict of interest

The authors declare that they have no conflict of interest.

## Supplementary data

Supplementary data is available at *Horticulture Research* online.

## Supplementary Material

Web_Material_uhad013
